# Utilization of paramagnetic relaxation enhancements for structural analysis of actin-binding proteins in complex with actin

**DOI:** 10.1038/srep33690

**Published:** 2016-09-22

**Authors:** Shuxian Huang, Ryo Umemoto, Yuki Tamura, Yutaka Kofuku, Taro Q. P. Uyeda, Noritaka Nishida, Ichio Shimada

**Affiliations:** 1 Graduate School of Pharmaceutical Sciences, The University of Tokyo, Hongo, Bunkyo-ku, Tokyo 113-0033, Japan; 2 Biomedicinal Information Research Center, National Institute of Advanced Industrial Science and Technology (AIST), Aomi, Koto-ku, Tokyo 135-0064, Japan

## Abstract

Actin cytoskeleton dynamics are controlled by various actin binding proteins (ABPs) that modulate the polymerization of the monomeric G-actin and the depolymerization of filamentous F-actin. Although revealing the structures of the actin/ABP complexes is crucial to understand how the ABPs regulate actin dynamics, the X-ray crystallography and cryoEM methods are inadequate to apply for the ABPs that interact with G- or F-actin with lower affinity or multiple binding modes. In this study, we aimed to establish the alternative method to build a structural model of G-actin/ABP complexes, utilizing the paramagnetic relaxation enhancement (PRE) experiments. Thymosin β4 (Tβ4) was used as a test case for validation, since its structure in complex with G-actin was reported recently. Recombinantly expressed G-actin, containing a cysteine mutation, was conjugated with a nitroxyl spin label at the specific site. Based on the intensity ratio of the ^1^H-^15^N HSQC spectra of Tβ4 in the complex with G-actin in the paramagnetic and diamagnetic states, the distances between the amide groups of Tβ4 and the spin label of G-actin were estimated. Using the PRE-derived distance constraints, we were able to compute a well-converged docking structure of the G-actin/Tβ4 complex that shows great accordance with the reference structure.

Actin is a major cytoskeletal protein within eukaryotic cells. The polymerization and depolymerization of actin between the monomeric (G-actin) and filamentous (F-actin) forms are essential for the actin dynamics that control cell morphology and cell motility. These actin dynamics are spatiotemporally well-regulated by a wide variety of actin binding proteins (ABPs) that mediate diverse functions, including (1) sequestering the G-actin from polymerization, (2) facilitating the nucleation and elongation of actin filaments, (3) severing actin filaments, and (4) bundling or branching of actin filaments to form higher order structures^1^,^2^. Therefore, the structural analyses of the interactions between actin and ABPs are essential, in order to understand the mechanisms by which ABPs modulate actin dynamics.

Although numerous G-actin and ABP complex structures have been determined by X-ray crystallography^3^, the crystallization of G-actin/ABP is not always successful, especially because high salt concentrations induce the polymerization of G-actin, thus precluding the growth of three-dimensional crystals. Structural analyses of the F-actin and ABP complexes using cryoEM have also been hampered by the inherent flexibility and polymorphism of the F-actin structure^4^. Although recent technical advances in the cryoEM fields have yielded the near-atomic resolution structures of F-actin alone^5^,^6^,^7^, obtaining structures at sufficient resolution to interpret the interaction between F-actin and ABP on the single-amino-acid level is still challenging^7^. Moreover, the X-ray crystallography and cryoEM methods both face difficulties when the ABPs interact with G- or F-actin with low affinity or multiple binding modes^8^.

The NMR method has been utilized to investigate the interactions between G-actin and ABPs with both strong and weak binding affinities, and provided information concerning the conformational changes of ABPs upon binding to G-actin, and the G-actin binding sites on ABPs^9^,^10^,^11^,^12^. However, the NMR analyses of the ABP binding site on G-actin are difficult because actin cannot be expressed by *E.coli*, and instead requires a eukaryotic expression system^13^,^14^,^15^. Although a recent study succeeded in the NMR observation of the sidechain methyl group of G-actin expressed in *Pichia pastoris*^16^, it is impossible to determine the accurate 3D structure of the G-actin/ABP complex, due to lack of intermolecular distance information between the ABP and G-actin.

An alternative NMR approach for the structure determination of a large molecular weight complex is the paramagnetic relaxation enhancement (PRE) method. Since the unpaired electron of a nitroxyl radical, such as MTSL ((1-Oxyl-2,2,5,5-tetramethyl-∆3-pyrroline-3-methyl) Methanethiosulfonate), causes line broadening of the NMR signals in a distance-dependent manner up to 20 Å, the long-range distance information can be obtained from the PRE experiments of a protein complex containing a spin label at a specific site^17^. The PRE-based distant constraints have been frequently used for structure determination and validation of proteins and protein-ligand complexes^18^,^19^,^20^,^21^,^22^. In the case of the G-actin/ABP complex, the PRE-derived intermolecular distance information would be obtained by observing the NMR signals of ABP in complex with G-actin containing a nitroxyl spin label at a selected position.

In this study, we developed the PRE method for the structural analysis of the G-actin/ABP complexes, using G-actin and thymosin β4 (Tβ4) as a model system. Tβ4 is a 43 residue actin-binding protein. Based on previous NMR and X-ray crystallographic studies, it was demonstrated that Tβ4 adopts an elongated conformation in the G-actin bound state, and forms two amphipathic helices at the N- and C-termini^10^,^12^,^23^. These N- and C-terminal helices bind to the barbed end and the pointed end of G-actin, respectively, thereby inhibiting the G-actin monomer from polymerizing into existing actin-filaments. Recently, the crystal structure of full length Tβ4 in complex with G-actin was solved, by using the fusion construct of Tβ4 connected to *Pichia pastoris* actin^24^. Therefore, we considered the Tβ4/G-actin complex to be an appropriate target to evaluate the feasibility of the PRE-based method.

To perform the PRE experiment, we produced recombinant human β-actin that contains a cysteine mutation for the site specific spin labeling, by using the baculovirus insect cell expression system. Using the spin labeled actin, we performed PRE experiments and collected intermolecular distance information between G-actin and ABP, based on the line-broadening of the NMR signals of ABP. Finally, we computed the docking structures of the actin/ABP complex, using the intermolecular distance information as distance constraints.

## Results

### Characterization of the recombinant actin

Recombinant human β-actin was prepared using the baculovirus expression system. The β-actin construct was designed, according to the strategy developed by Noguchi *et al*., in which Tβ4 was fused to the C-terminus of the β-actin^25^. This construct enables the purification of recombinant actin that is free of endogenous actin of the host cells. We confirmed that the recombinant β-actin expressed by insect cells retains the polymerization and depolymerization activities by ultracentrifugation (Supplementary Fig. 1a) and the similar secondary structure composition compared to the rabbit α-actin by the circular dichroism analysis (Supplementary Fig. 1b). The dissociation constant of recombinant β-actin for Tβ4 was 0.5 ± 0.1 μM from the ITC analyses (Supplementary Fig. 2a), which was consistent with the previously estimated binding constant using platelet actin^26^. The ^1^H-^15^N HSQC spectrum of ^15^N-labeled Tβ4 in complex with recombinant β-actin was similar to that obtained in the previous NMR study of Tβ4 bound to α-actin (Supplementary Fig. 3a)^9^. The slight difference between two spectra may reflect the differences in the amino acid sequence between rabbit α-actin and human β-actin. Based on these results, we concluded that the recombinant β-actin is structurally and functionally equivalent to the native β-actin.

### Design and characterization of β-actin spin-labeled at a specific position

To make a site-specific spin-labeled actin, the endogenous cysteine residues that were exposed to the solvent were substituted with alanine, namely C272A/C374A (hereafter called actin-2A). We confirmed that the actin-2A did not react with the spin-labeling reagent, IASL (3-(2-iodoacetamido-2,2,5,5,tetramethyl-1-pyrrolidinyloxy radical (iodoacetamido-PROXYL))), while the wild type β-actin reacted with two IASL molecules. The ITC analyses showed that the actin-2A mutant retains almost equivalent Tβ4-binding activity to the wild type β-actin, with a dissociation constant of 0.3 ± 0.1 μM (Supplementary Fig. 2b). The ^1^H-^15^N HSQC spectrum of Tβ4 bound to actin-2A was similar to that of the wild type β-actin, except for minor chemical shift changes at the N-terminal residues presumably due to the mutation at C374, which is proximal to these residues in the model structure (Supplementary Fig. 3b). Therefore, we used the actin-2A mutant for the following PRE experiments.

**Figure 1:**
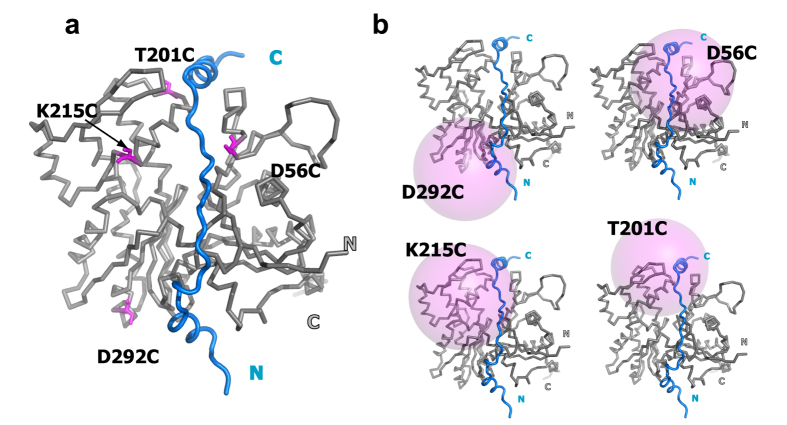
Design of β-actin mutants for PRE experiments. (**a**) The model structure of the human β-actin/Tβ4 complex is drawn by a ribbon and tube diagram, colored grey (G-actin) and blue (Tβ4), respectively. This structure was built based on the X-ray structure of the *Pichia* actin/Tβ4 complex (PDB code: 4PL7). The positions of 4 residues (D56, T201, K215 and D292) that were mutated to cysteine for IASL labeling are shown by magenta sticks. (**b**) Estimation of the range that could be affected by PRE. Pink spheres indicate the regions within 20 Å from the Sγ atoms of D56C, T201C, K215C and D292C.

Using actin-2A as a template, we introduced a single cysteine mutation at D56, T201, K215, and D292 for the site-specific IASL labeling (Fig. 1a). These residues were selected for site-specific IASL labeling for the following reasons. Firstly, the Tβ4 residues are located within 20 Å from these residues in the model structure of the actin/Tβ4 complex, and thus the PRE effect would be observed (Fig. 1b). Secondly, the solvent accessibilities of the residues are sufficiently high, in order to conjugate the spin label efficiently (D56:54.4%, T201:83.9%, K215:59.1% and D292:56.9% for side chain atoms). These actin mutants were expressed and purified as described in the Methods section. The yields and polymerization activities of actin mutants were summarized in Supplementary Table 1.

Prior to the PRE experiments, each cysteine mutant was treated with IASL, and the labeling efficiency was examined by the mass spectrometry. The mass increase of the mutants indicated that all of the mutants were labeled with IASL with high efficiency (60–80%). We confirmed that the ^1^H-^15^N HSQC spectra of Tβ4 in complex with these IASL-labeled actin mutants were similar to that in the complex with actin-2A, indicating that the Tβ4-binding mode of actin was not affected by introducing the IASL spin label.

### PRE experiments between spin-labeled recombinant actin and thymosin β4

To obtain the intermolecular distance constraints, we performed PRE experiments between spin-labeled recombinant actin and Tβ4. For the PRE experiments, 100 μM of ^15^N-labeled Tβ4 was mixed with 170 μM of IASL-labeled β-actin, and the ^1^H-^15^N HSQC spectrum was acquired in the paramagnetic states. After adding 2 mM ascorbic acid, the ^1^H-^15^N HSQC spectrum was acquired in the diamagnetic states.

**Figure 2:**
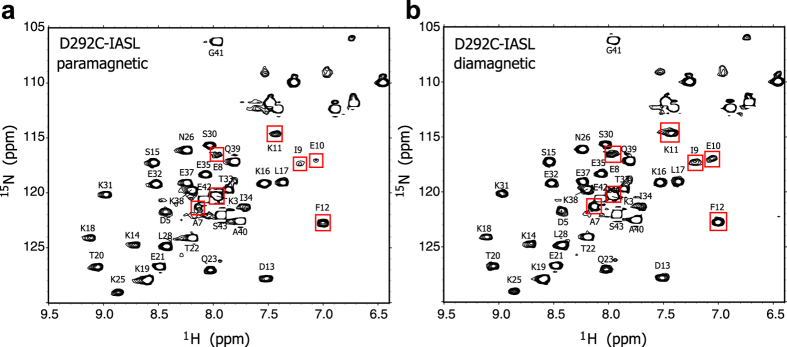
PRE experiments between Tβ4 and D292C-IASL actin. ^1^H-^15^N HSQC spectra of Tβ4 in complex with D292C-IASL actin in the paramagnetic (**a**) and diamagnetic (**b**) states. Assignments of Tβ4 signals in the complex with G-actin were based on the previous report^9^. Amide resonances that showed significant intensity reductions (I_para_/I_dia_ < 0.70) are indicated by red boxes.

**Figure 3:**
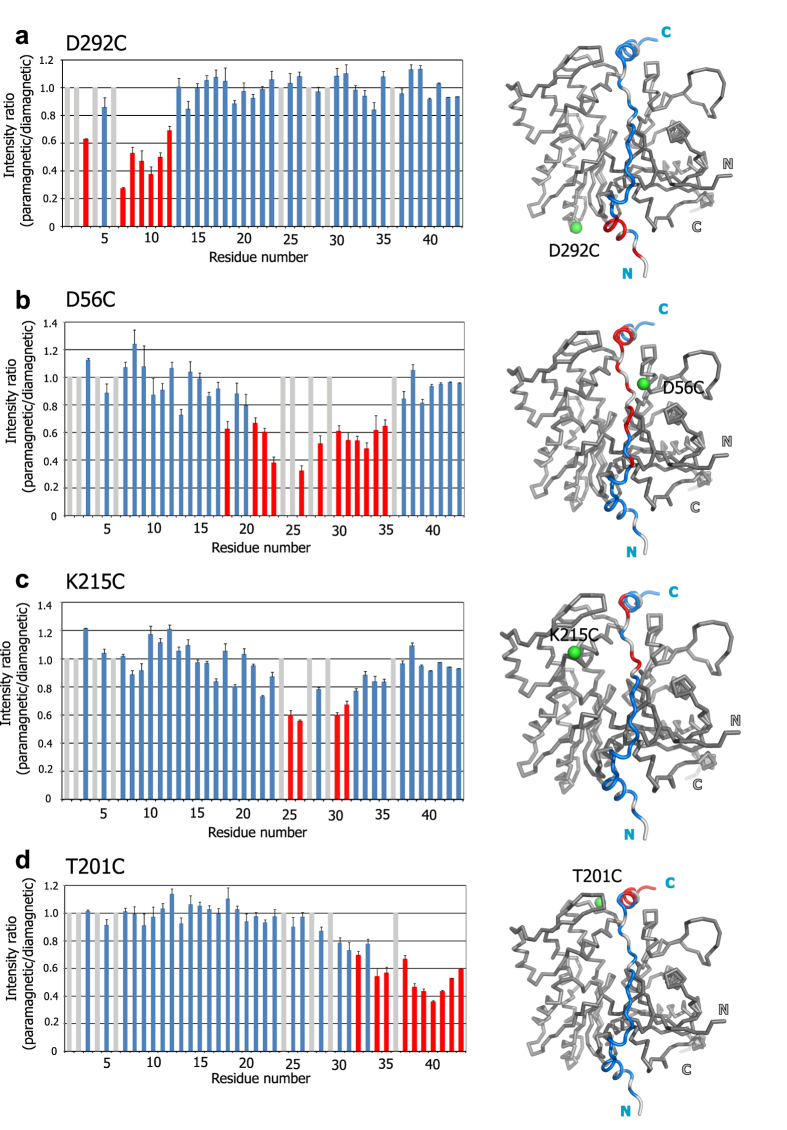
Results of PRE experiments between IASL labeled actin and Tβ4. (**a–d**), Left, plots of intensity ratio (I_para_/I_dia_) of Tβ4 signals against its residue number are shown, for Tβ4 in complex with β-actin modified with IASL at D292C (**a**), D56C (**b**), K215C (**c**), and T201C (**d**). The residues with grey bars are those with no data, due to the overlapping resonances or perturbed resonances upon IASL modification. The error bars were calculated based on the signal-to-noise ratios. Bars corresponding to the residues with significant intensity reductions (I_para_/I_dia_ < 0.70) are colored red. Right, mapping of the affected residues in the PRE experiment on the model structure of the human β-actin/Tβ4 complex. In the Tβ4 molecule, the affected residues are colored red, while unaffected residues and those with no data are colored blue and grey, respectively. The actin molecule is depicted by grey ribbon diagram, and the Sγ atom of the spin-labeled residue is shown by a green sphere.

Figure 2 shows the ^1^H-^15^N HSQC spectra of Tβ4 in complex with D292C-IASL actin in the paramagnetic and diamagnetic states. In the paramagnetic state (Fig. 2a), some amide signals showed significant intensity reductions, as compared with those in the diamagnetic state (Fig. 2b). We calculated the intensity ratios between the paramagnetic and the diamagnetic states (I_para_/I_dia_) for each resonance and mapped the residues with significant intensity reductions (I_para_/I_dia_ < 0.70) on the structure of the actin/Tβ4 complex (Fig. 3a). The mapping showed that the affected residues were clustered on the N-terminal side of Tβ4, which was adjacent to the introduced IASL at the position of D292C. Likewise, we performed the PRE experiments, using the IASL-labeled actin mutants D56C, K215C, and T201C (Fig. 3b–d). The residues with intensity ratios (I_para_/I_dia_) below 0.70 were clustered in the position adjacent to the introduced IASL in each experiment. These results indicated that the observed intensity reductions reflect the PRE effect derived from the introduced spin label, and the proximal residue pairs in the actin/Tβ4 complex can be identified by using spin-labeled actin.

### Building a model structure of the actin/Tβ4 complex using distance constraints from PRE

Finally, we built a model structure of the actin/Tβ4 complex, based on the distance information obtained from the PRE experiments. For the docking model calculation using HADDOCK^27^, the distances between the amide group and the paramagnetic center were calculated for 33 residues with significant intensity reductions (I_para_/I_dia_ < 0.7), as described in the Methods and summarized in Supplementary Table 2. For the HADDOCK calculation, the isolated coordinates of human β-actin and Tβ4, which were built based on the *Pichia* actin/Tβ4 hybrid structure, were used as the initial model. 1,000 complex structures were generated in the first round of the rigid body docking, and 200 structures were selected in the semi-flexible docking and the water refinement steps.

**Figure 4:**
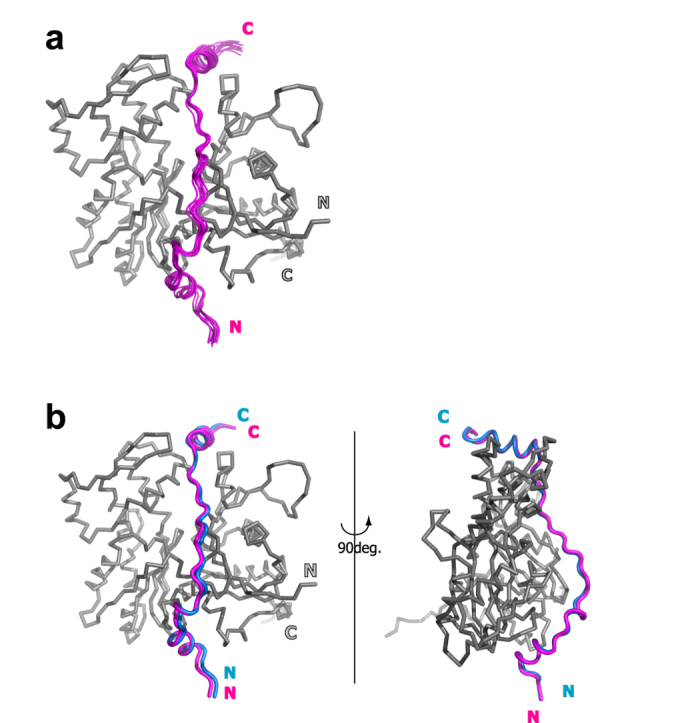
Model structures of the Tβ4/G-actin complex. (**a**) Superimposition of the 10 lowest energy models of the Tβ4/G-actin complex. The actin molecule is depicted by grey ribbon diagram, and the Tβ4 molecule is colored magenta. The structural alignment was done using the G-actin moiety. (**b**) Superimposition of the mean structure of the 10 lowest energy models (magenta) and the X-ray derived model structure (blue). The structural alignment was done using the G-actin (grey) moiety.

Among the obtained 200 structures, the 10 structures with the lowest energy were well-converged, with an average backbone RMSD to mean of 0.74 ± 0.17 Å (Fig. 4a). Furthermore, the mean structure of the 10 lowest energy models exhibited great accordance with the X-ray based model structure, with the backbone RMSD of 0.89 Å (Fig. 4b). Based on this result, we concluded that a model structure of the actin/Tβ4 complex can be determined by utilizing the distance constraints obtained from PRE experiments.

## Discussion

In this study, we established a method to obtain intermolecular distance information between G-actin and ABP, based on a paramagnetic relaxation enhancement (PRE) experiments. To perform the PRE experiments, G-actin mutants with a single reactive cysteine residue were prepared by the baculovirus expression system. The residues showing significant intensity reductions in the PRE experiments were identified as the residues proximal to the introduced spin label in the complex, and used for the distance constraints for docking calculations. The mean structure obtained from the docking models based on the PRE-derived constraints coincided well with the recently solved X-ray structure of Tβ4/G-actin, with a backbone RMSD of 0.89 Å. Therefore, this study is the first demonstration that the structure of the G-actin/ABP complex can be modeled based on the PRE-derived distance constraints.

In the present study, we successfully built the docking model of Tβ4/G-actin, by using 33 unambiguous constraints from four spin-labeled mutants (D56C, T201C, K215C and D292C). To evaluate the contributions of each spin labeling site to the convergence of the model structures, we calculated the docking model by using a reduced number of constraints (Supplementary Figs 4 and 5). The results revealed that the exclusion of the distance constraints derived from either D292C or T201C, which is close to the N- or C-terminus of Tβ4 in the complex, respectively, significantly affected the convergence of the docking model (Supplementary Fig. 4). In addition, the constraints from only those two spin labeling sites using T201C and D292C also failed to build the converged docking model (Supplementary Fig. 5). Therefore, in the case of G-actin/Tβ4 complex, three spin labeling sites, including T201C, D292C, and either D56C or K215C, are necessary to build a converged docking model. In terms of accuracy, the backbone RMSDs between the mean of the 10 lowest energy models based on three spin labeling sites and the X-ray based model structure were 1.02 Å (for T201C, D292C and D56C) and 1.54 Å (for T201C, D292C, and K215C). By using all four spin labeling sites, the backbone RMSD between the mean and X-ray based model structures further improved to 0.89 Å.

How would the cysteine positions for spin labeling be selected if the information regarding the ABP binding site on actin is not available? In such cases, it would be necessary to test numerous actin proteins spin labeled at different positions to observe the PRE effect on ABPs. To estimate how many mutants need to be tested, we calculated the surface exposed residues in actin that can be affected by each spin labeling site, assuming the range of the PRE effect has 20 Å in radius. Although any solvent exposed residues in actin can be used for the site specific spin labeling, the four spin labeled actin used in this study (i.e. D56C, T201C, K215C, D292C) can cover 57% of the solvent exposed residues of actin (Fig. 1b). By utilizing three additional mutants (for example, Q121C, C272, and Q354C), the percentage of the coverage could be further extended to 90.4%, which would be sufficient to find the appropriate spin labeling sites for PRE observation.

In this study, the distance information were calculated based on the intensity ratio of the HSQC signals in the paramagnetic and diamagnetic states (I_para_/I_dia_), instead of the multiple points R2 curve fitting experiments, due to the low long-term stability of the G-actin/Tβ4 sample. Since the single time point experiment is less quantitative^28^, we set the relatively large error range for the distance constraints (±5.0 Å). To evaluate whether the distance restraints and the error range were appropriate, we measured the distances between the Sγ of spin labeled residue in actin and amide protons of Tβ4 in the docking model and judged whether the distance constrains were satisfied or violated. As shown in Supplementary Tables 2, 29 out of 33 atom pairs were placed within the upper and lower limits of the distance restraints, indicating that the distance restraints and the error range used in this study were appropriate. Therefore, although the distance constrains determined from PREs measured by single time-point experiment harbor a large error range, the inclusion of a large number of distance constraints by increasing the spin labeling sites would improve the convergence and accuracy of the structures of actin and ABPs. A recent study also demonstrated that the convergence of the protein structure was significantly improved by the usage of a considerable number of the PRE-derived constraints, in a case where the NOE derived distance information is limited^29^. We also suggest that the more accurate transverse relaxation rates would be measured for G-actin/Tβ4 complex, by employing the methyl-TROSY-based technique^30^. Such analyses will allow us to retrieve more accurate distance information of actin/ABP complexes, and may identify the lowly populated conformations, if any^31^,^32^.

Sequences homologous to Tβ4 are widely found in many actin-binding proteins, and are known as β-thymosin (βT) or WASP homology 2 (WH2) motifs^33^. Although many crystal structures have been reported for the βT/WH2 motifs in the G-actin bound form, the entire binding mode remains elusive, due to lack of the electron density of the C-terminal region in the crystal structure. It is known that G-actin fluctuates between multiple conformations at the pointed-end side by the movement of sub-domains 2 and 4, which form a binding site for the C-terminal segment of the βT/WH2 motifs^24^. Due to this conformational flexibility, the interactions of G-actin with the C-terminal region of the βT/WH2 domains are unstable, and thus making the crystallographic approach difficult. In contrast, our PRE method can analyze the interactions of G-actin and ABPs in solution, and thus would be advantageous for investigating the dynamical binding modes of βT/WH2 motifs in the G-actin bound state.

The PRE-based method would also be applicable to the interactions between ABPs and F-actin, which are difficult to analyze by the X-ray crystallography or cryoEM methods. If ABP undergoes fast exchange between the unbound and bound states on the NMR time scale, then the PRE of ABPs in the F-actin bound state would be detected by observing the NMR signals of ABPs in the unbound state. It should be noted that, to perform the PRE experiment for the F-actin/ABP complex, it is necessary to design a spin-labeled actin that will not affect the polymerization to F-actin. In fact, among the four cysteine mutants used in this study, only the D292C mutant retains the polymerization activity similar to that of wild type actin (Supplementary Table 1). The recently solved high resolution EM structure of the F-actin would be useful for determining such mutants^5^,^6^,^7^,^34^,^35^. In addition, the actin-binding site of the ABPs could be identified by using the transferred cross-saturation method^36^,^37^,^38^. This information would be useful for improving the convergence and accuracy of the complex structure.

## Methods

### Expression and Purification of thymosin β4

Non-labeled or uniformly^15^N-labeled Tβ4 protein was expressed in *E. coli* as a GST-fusion protein and was purified by GST affinity chromatography. After cleavage and removal of the GST-tag, the protein was further purified by reverse phase chromatography. The purified protein was lyophilized, and then dissolved in 5 mM Tris-HCl, pH 7.8, 0.1 mM CaCl_2_. The stock solutions were concentrated with an Amicon Ultra filter (Millipore) when necessary.

### Construction of recombinant baculoviruses

The cDNA encoding β-actin was amplified from a human liver cDNA library (TaKaRa) by PCR. In addition, the linker (ASRGGSGGSGGSA), Tβ4, and octahistidine tag were attached to the C-terminus of the β-actin sequence, according to the previously established pTIKL-ART vector^25^, and cloned into the baculovirus transfer vector pFastBac1 (Invitrogen). To enhance the expression level, the L21 bp (AACTCCTAAAAAACCGCCACC) sequence was inserted upstream of the start codon^39^. The cysteine mutants of β-actin were generated according to the Quik Change mutagenesis protocol.

### Expression and Purification of human β-actin and its mutants

Human β-actin was expressed in expresSF+ insect cells (Protein Sciences) by infection with the amplified recombinant baculoviruses encoding human β-actin, at a multiplicity of infection of 4 for each baculovirus. After an incubation at 27 °C for 48 hours, the cells were harvested and resuspended in lysis buffer (10 mM Imidazole, 10 mM HEPES, pH 7.4, 300 mM NaCl, 2 mM MgCl_2_, 1 mM ATP, 7 mM 2-mercaptoethanol, 4% TritonX-100, 1 mg/mL Tween-20) containing 1 × protease inhibitor mixture (EDTA free) (Nacalai Tesque). The purification was performed at 4 °C, according to the method described previously^25^. First, the cell suspension was sonicated and clarified by centrifugation at 36,000 g for 60 min, and the supernatant thus obtained was purified using HIS-Select Nickel Affinity Gel (Sigma). The C-terminal tag was cleaved by chymotrypsin, followed by further purification using a Hi-Trap Q HP column (GE Healthcare). The fractions containing intact actin were polymerized by adding 50 mM KCl and 1 mM MgCl_2_ (final concentrations), and ultracentrifuged (80,000 rpm for 30 min). The pellet was suspended in G-buffer (5 mM Tris-HCl, pH 7.8, 0.1 mM CaCl_2_, 0.2 mM DTT, 0.2 mM ATP, 0.01% NaN_3_), dialyzed against G-buffer overnight, and then clarified by ultracentrifugation to obtain the recombinant G-actin in the supernatant.

### ITC analyses

The binding constants for the actin-Tβ4 interaction were measured by ITC, using a VP-ITC (MicroCal) instrument at 25 °C. The protein samples were dialyzed against G-buffer. The titration of the actin with Tβ4 was performed by 29 injections of 10 μl of the Tβ4 solution (100 μM) at intervals of 240 s into a sample cell containing actin (10 μM). The data were analyzed with VPView2000 ITC software.

### Site-directed spin labeling of β-actin

For site-specific spin labeling, four cysteine mutants of β-actin, D56C, T201C, K215C and D292C, were prepared, based on actin-2A (C272A/C374A). After purification, the cysteine mutants were dialyzed against G-buffer (-DTT) overnight, to remove DTT. After dialysis, the proteins were treated with the spin-label reagent IASL (3-(2-iodoacetamido-2,2,5,5,tetramethyl-1-pyrrolidinyloxy radical (iodoacetamido-PROXYL))) (Toronto Research Chemicals) at a molar ratio of 30 : 1 (IASL:actin), for 17 hours at 4 °C in the dark. After the reaction, the proteins were dialyzed against G-buffer (-DTT) overnight, to remove the unreacted IASL. The efficiency of the spin-labeling reaction was examined by MALDI-TOFMS, and the amount of spin-labeled actin was estimated on the basis of the increase of mass as compared to the actin without IASL treatment.

### NMR spectroscopy

Spin-labeled actin samples were dialyzed against NMR buffer (5 mM Tris-HCl, pH 6.9, 0.1 mM CaCl_2_, 1 mM ATP) overnight and the^15^N labeled Tβ4 stock solutions were added to the actin solution, and then concentrated with an Amicon Ultra filter (Millipore). The final concentrations of Tβ4 and G-actin were 100 μM and 170 μM, respectively. D_2_O and NaN_3_ were added to the NMR sample (final concentrations, 10% and 0.01%, respectively) and 0.3 ml of the sample solution was transferred to a 5 mm Shigemi tube for NMR measurements.

The ^1^H-^15^N HSQC spectra were acquired at 25 °C with a Bruker Avance 800 MHz spectrometer equipped with a cryogenic probe. To collect the spectrum of the diamagnetic state, 2 mM of ascorbic acid was added to the sample and incubated for 0.5 hour at 4 °C before NMR measurements.

### NMR analyses

All spectra were processed by the Bruker Topspin 2.1 software, and the data were analyzed by Sparky (T. D. Goddard and D. G. Kneller, Sparky 3, University of California, San Francisco, CA). The error bars were estimated based on the signal-to-noise ratio calculated by the Sparky software. The residues with overlapping resonances were omitted from the analyses. Intensity ratios (I_para_/I_dia_) were normalized by averaging the values for amides >30 Å away from the Sγ atom of the spin-labeled cysteine, as determined from the model structure of the actin/Tβ4 complex.

### Calculations of the actin-Tβ4 complex structure

The model structure of Tβ4 bound to human β-actin was constructed based on the X-ray structure of *Pichia* actin and Tβ4 (PDB code : 4PL7), using the program MODELLER^40^. The amino acid sequence identity of *Pichia* actin and human β-actin is 85.1%.

The structures of the actin/Tβ4 complex, based on the PRE-derived distance constraints, were calculated by using the HADDOCK program^27^. The model structure of the complex was separated into the individual human β-actin and Tβ4 coordinates, and used as the initial structures for the HADDOCK calculations. For the model structure of human β-actin, four cysteine mutations (D56C, T201C, K215C and D292C) were introduced by using the PyMOL Molecular Graphics System (version 1.7.2.1, Schrödinger, LLC). The distance between the amide group of Tβ4 and the paramagnetic center of the nitroxyl radical of spin-labeled actin was calculated, according to the method described by Battiste and Wagner^17^. The PRE-derived distance constraints were set between the amide protons and the Sγ atoms of the mutated cysteine residues, with the error range of 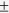
5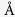 (Supplementary Table 2).

In total, 1,000 complex structures were calculated in the rigid body docking and, 200 structures with low energy scores were selected for the following round of the semi-flexible docking and the water refinement procedures. We tested both 0.8 and 0.7 cutoff of I_para_/I_dia_ for the distance restraints, resulting in the slightly better convergence for 0.7 cutoff than 0.8 cutoff (with RMSD 0.74 ± 0.17 Å vs 0.80 ± 0.16 Å, respectively) and the better accuracy (RMSD for reference crystal structure 0.89 Å vs 0.95 Å). For this reason, we used the threshold of 0.7. The structures were drawn using the PyMOL Molecular Graphics System.

## Additional Information

**How to cite this article**: Huang, S. *et al*. Utilization of paramagnetic relaxation enhancements for structural analysis of actin-binding proteins in complex with actin. *Sci. Rep.* **6**, 33690; doi: 10.1038/srep33690 (2016).

## Supplementary Material

Supplementary Information
