# Supplementary material for: Utilization of paramagnetic relaxation enhancements for structural analysis of actin-binding proteins in complex with actin
Source: Sci Rep. 2016 Sep 22;6:33690. doi: 10.1038/srep33690 (PMC5031973; doi:10.1038/srep33690)
Supplement: Supplementary Information [file srep33690-s1.pdf]

Supplementary information to:

Utilization of paramagnetic relaxation enhancements for structural analysis of actin-binding proteins in complex with actin

Shuxian Huang[1, 3], Ryo Umemoto[1, 3], Yuki Tamura[1], Yutaka Kofuku[1], Taro Q.P. Uyeda[2, 4], Noritaka Nishida[1], and Ichio Shimada[1, 2]*

[1] Graduate School of Pharmaceutical Sciences, The University of Tokyo, Hongo, Bunkyo-ku, Tokyo 113-0033, Japan

[2] Biomedicinal Information Research Center, National Institute of Advanced Industrial Science and Technology (AIST), Aomi, Koto-ku, Tokyo 135-0064, Japan

[3] Equal contributions

[4] Present address: Department of Physics, Faculty of Science and Engineering, Waseda University, Okubo, Shinjuku-ku, Tokyo 169-0072, Japan

*Corresponding should be addressed: shimada@iw-nmr.f.u-tokyo.ac.jp

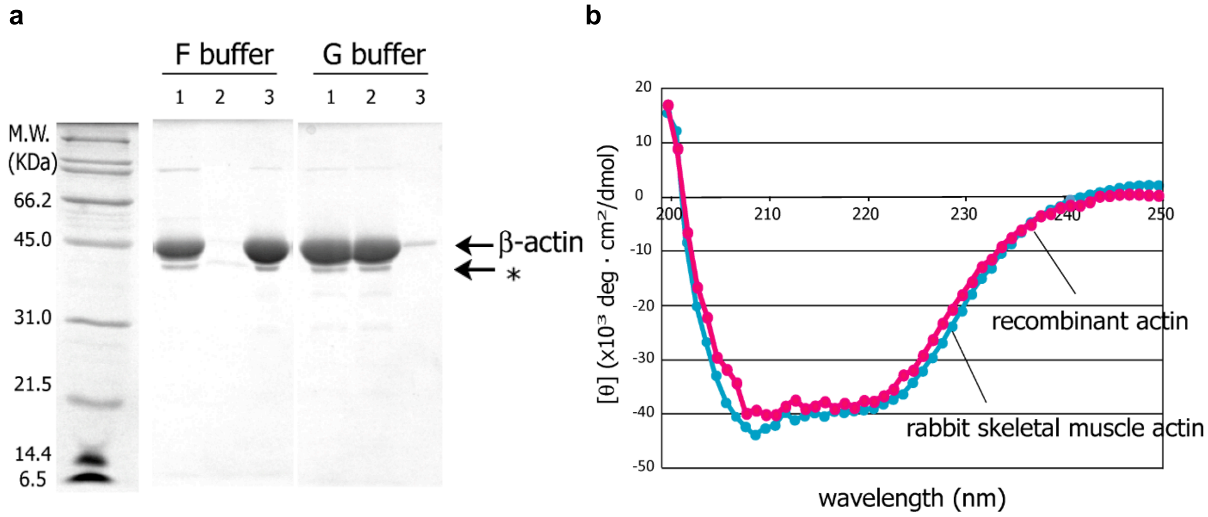

Supplementary Figure 1　Biophysical characterizations of recombinant actin

(a) The polymerization and depolymerization activities of the purified β-actin were confirmed by ultracentrifugation. The SDS-PAGE analysis of β-actin in F and G-buffers before ultracentrifugation (lane 1), and in the supernatant (lane 2) and pellet (lane 3) fractions after ultracentrifugation (80,000 rpm, 30 min) is shown.　SDS-gels were stained with Coomassie Brilliant Blue.　In F-buffer (10 mM Tris-HCl, pH 7.2, 100 mM KCl, 2 mM $MgCl_2$, 1 mM DTT, 0.5 mM ATP), actin appeared in the pellet fraction. In contrast, in G-buffer, actin appeared in the supernatant fraction.　This result indicates that the purified recombinant human β-actin has the proper polymerization and depolymerization activities.　The minor band shown by asterisk correspond to the actin nicked at the flexible segment (presumably the DNase I binding loop a.a.38-52) by chymotrypsin digestion. As shown in the SDS-gel, this nicked actin still has the polymerization and depolymerization activities, indicating that it retains structural integrity.　A similar digested product was used and

shown to retain the Tβ4-binding activity in the previous study (Domanski M et al. JBC(2004)279, 23637-45). (b) The circular dichroism (CD) spectra of the recombinant human β-actin (magenta) and the rabbit α-actin (cyan, purchased from Cytoskeleton). The CD experiments were performed in G-buffer at 25 ºC. The protein concentrations were both 6.7 μM.

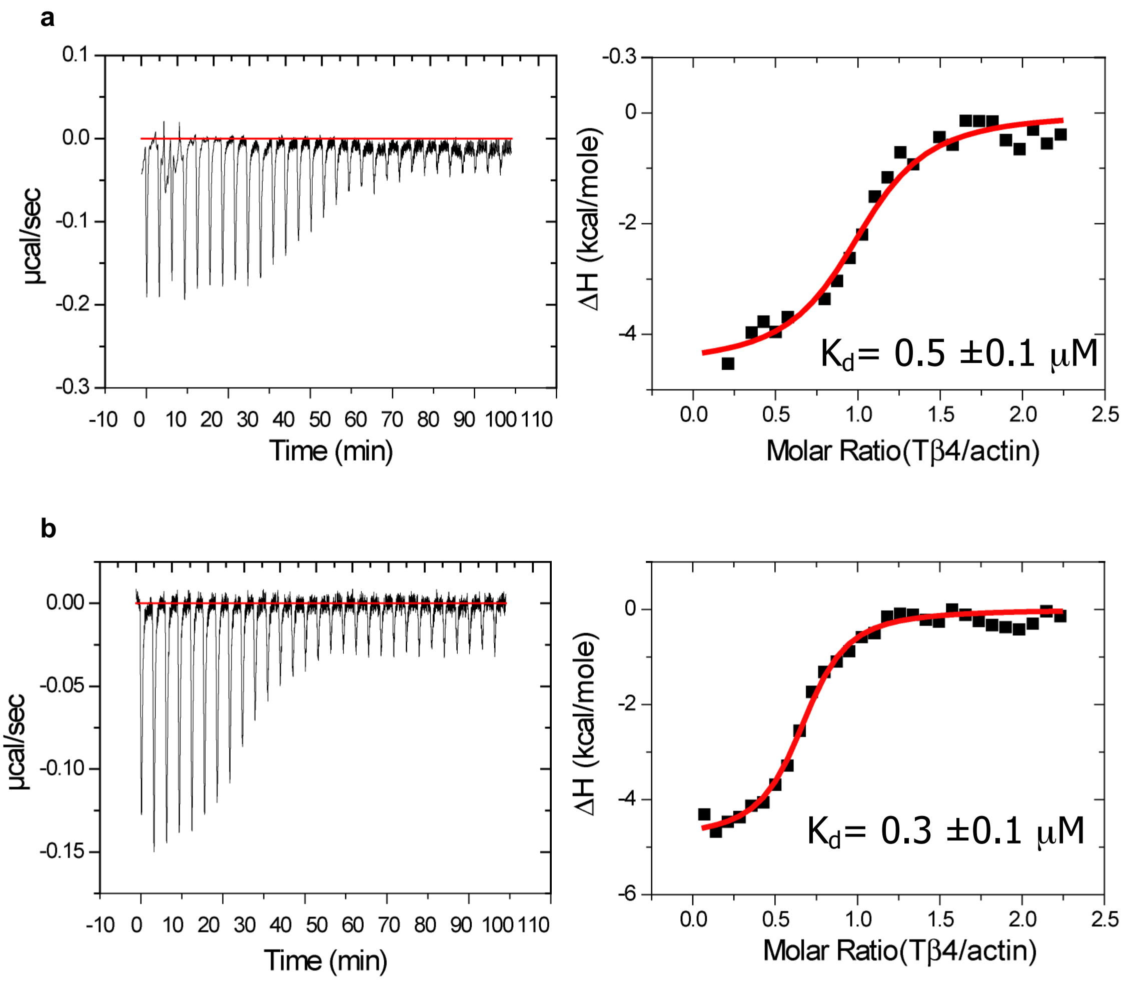

<u>Supplementary Figure 2　ITC experiments for the thymosin β4 and recombinant β-actin</u>

The raw data (left) and the binding isotherms (right) of ITC experiments for (a) Wild type β-actin, (b) actin-2A mutant (C272A/C374A).　Kd values were estimated by one-site binding model.

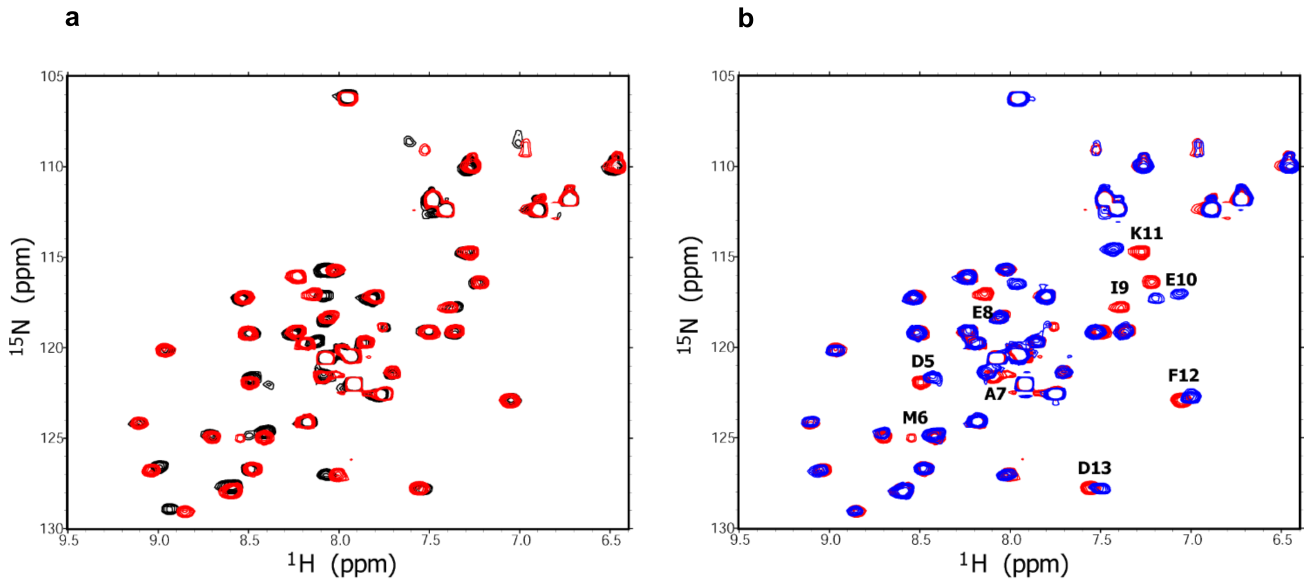

Supplementary Figure 3 ^{1}H-^{15}N HSQC spectra of Tβ4 in complex with α-actin, recombinant human β-actin and actin-2A

(a) Superimposition of ^{1}H-^{15}N HSQC spectra of Tβ4 in complex with α-actin (black) and recombinant human β-actin (red). (b) Superimposition of ^{1}H-^{15}N HSQC spectra of Tβ4 in complex with recombinant human β-actin (red) and actin-2A (blue). Residues that showed chemical shift perturbations are indicated in the spectrum.

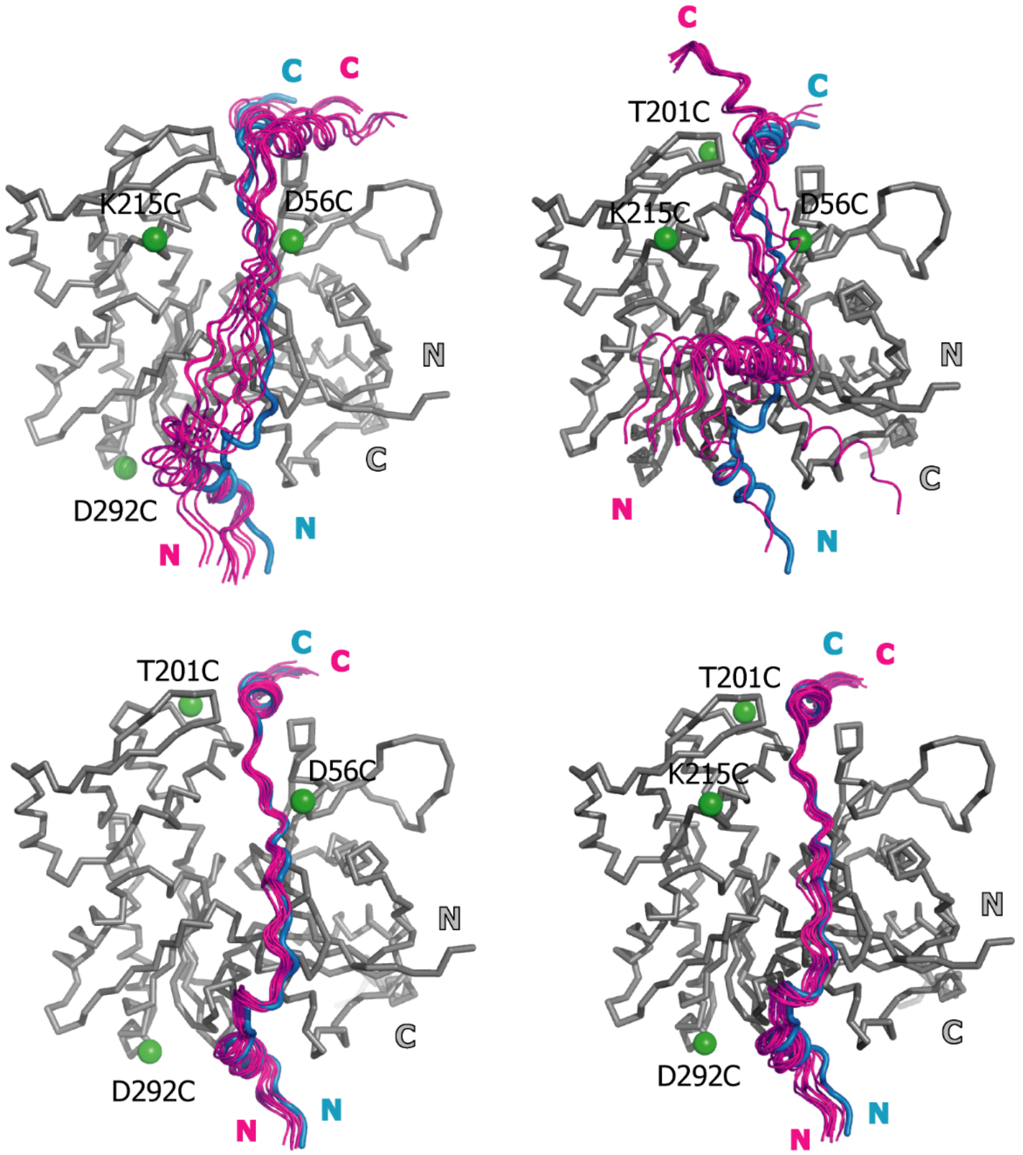

Supplementary Figure 4 Model structures of actin/Tβ4 based on the three spin labeling positions

Comparison of the X-ray based model structure of actin/Tβ4 (blue) and the superimposition of the 10 lowest energy models (magenta) based on the three spin labeling positions. The structural alignment was done using the G-actin moiety (grey). The Sγ atoms of the spin-labeled residues used in the structure calculation are shown in green spheres.

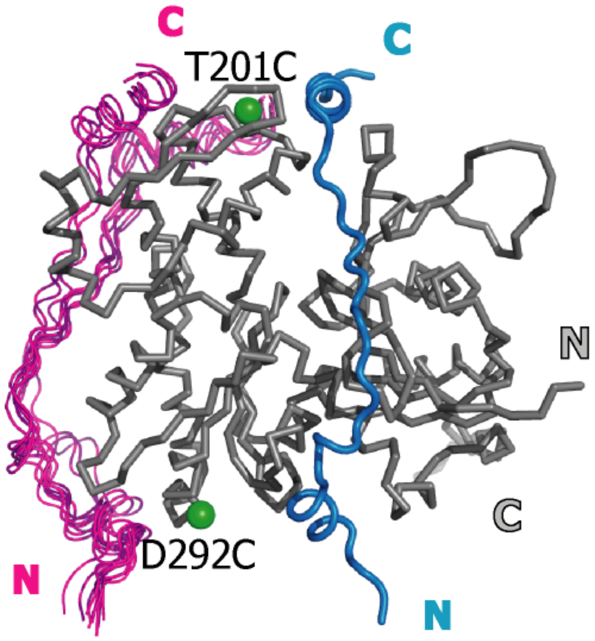

<u>Supplementary Figure 5 Model structures of actin/Tβ4 based on the two spin labeling positions</u>

Comparison of the X-ray based model structure of actin/Tβ4 (blue) and the superimposition of 10 lowest energy models (magenta) based on the two spin labeling positions. The structural alignment was done using the G-actin moiety (grey). The Sγ atoms of the spin-labeled residues used in the structure calculation are depicted by green spheres.

Supplementary Table 1 Yield and polymerization activity of each recombinant actin mutant

Construct	Yield (mg/L culture)	Polymerization activity
WT	22.0	High
Actin-2A	26.0	High
D56C	5.7	Low
T201C	12.0	Low
K215C	4.2	Low
D292C	24.0	High

Supplementary Table 2 Distance constraints based on intensity reductions in PRE experiments

Sγ atom	NH group	Intensity ratio	Distance constraints(Å)	Error range (Å)	Distance in the model (Å)	Results*[a]
D56C	K18	0.623	15.2	±5.0	24.9	V
D56C	E21	0.666	15.7	±5.0	17.0	S
D56C	T22	0.600	15.0	±5.0	14.4	S
D56C	Q23	0.378	13.1	±5.0	10.9	S
D56C	N26	0.322	12.7	±5.0	4.4	V
D56C	L28	0.518	14.2	±5.0	7.8	V
D56C	S30	0.608	15.1	±5.0	11.9	S
D56C	K31	0.542	14.5	±5.0	15.3	S
D56C	E32	0.538	14.4	±5.0	16.3	S
D56C	T33	0.481	13.9	±5.0	14.8	S
D56C	I34	0.615	15.1	±5.0	15.5	S
D56C	E35	0.643	15.4	±5.0	18.1	S
T201C	E32	0.694	16.0	±5.0	15.2	S
T201C	I34	0.540	14.4	±5.0	12.2	S
T201C	E35	0.566	14.7	±5.0	11.1	S
T201C	E37	0.665	15.7	±5.0	11.6	S
T201C	K38	0.462	13.8	±5.0	9.3	S
T201C	Q39	0.431	13.5	±5.0	10.3	S
T201C	A40	0.357	12.9	±5.0	10.7	S
T201C	G41	0.432	13.5	±5.0	11.9	S
T201C	E42	0.527	14.3	±5.0	13.8	S
T201C	S43	0.596	15.0	±5.0	17.1	S
K215C	K25	0.599	15.0	±5.0	17.2	S
K215C	N26	0.554	14.6	±5.0	15.9	S
K215C	S30	0.599	15.0	±5.0	17.9	S
K215C	K31	0.671	15.7	±5.0	18.8	S
D292C	K3	0.629	15.3	±5.0	23.0	V
D292C	A7	0.270	12.3	±5.0	15.6	S
D292C	E8	0.526	14.3	±5.0	17.5	S
D292C	I9	0.467	13.8	±5.0	16.8	S
D292C	E10	0.372	13.1	±5.0	15.0	S
D292C	K11	0.498	14.1	±5.0	16.7	S
D292C	F12	0.690	15.9	±5.0	19.0	S

*[a]: S(satisfied), V(violated)
